# Roles of transcriptional factor Snail and adhesion factor E-cadherin in clear cell renal cell carcinoma

**DOI:** 10.3892/etm.2013.1345

**Published:** 2013-10-14

**Authors:** JINQUAN CAI

**Affiliations:** Department of Urology, Fuzhou General Hospital of Nanjing Military Command of Chinese PLA, Fuzhou, Fujian 350025, P.R. China

**Keywords:** clear cell renal cell carcinoma, E-cadherin, Snail, epithelial-mesenchymal transition

## Abstract

The aim of this study was to investigate the roles of the transcription factor Snail and adhesion factor epithelial-cadherin (E-cadherin) in clear cell renal cell carcinoma (CCRCC) and evaluate their correlation with tumor pathological grading, clinical stage, invasion and metastases. The expression of Snail and E-cadherin protein in 69 samples of CCRCC tissue, 58 samples of para-cancerous mucosa and 10 samples of normal renal tissue were detected using the immunohistochemical streptavidin-peroxidase method. The positivity rate of Snail in CCRCC was 82.61%, which was significantly higher than that in para-cancerous mucosa (43.10%, P<0.001). The positivity rate of E-cadherin in CCRCC was 31.88%, which was significantly lower than that in para-cancerous mucosa (91.38%, P<0.001). The expression of E-cadherin and Snail correlated significantly with tumor differential degree, clinical stage and the depth of tumor invasion and distant metastasis (P<0.05). There was a negative correlation between the expression of E-cadherin and Snail in CCRCC. The overexpression of Snail and reduced expression of E-cadherin may be important biological markers for the invasion and metastasis of CCRCC. The combined detection of E-cadherin and Snail has far-reaching significance for the prediction of CCRCC invasion and metastasis.

## Introduction

Tumorigenesis, invasion and metastasis are complex processes involving several signaling pathways, cell factors and other tumor-causing factors, and are among the main causes of mortality. Tumor invasion and metastasis, in addition to the biological activity of kidney cell transformation and migration, currently receive considerable attention. Metastatic kidney cancer is common in patients, and the treatment of renal cell carcinoma is becoming increasingly difficult. Thus, kidney cancer metastasis and infiltration are focal points in kidney cancer research.

Biological treatments for kidney cancer have limited availability. *In vitro* studies on the side-effects of gene therapy are limited, and the effects of specific clinical treatments remain unclear. Epithelial cadherin (E-cadherin), which is present in epithelial cells, mediates the connections between calcium-dependent adhesion molecules. E-cadherin, which is considered to be the most important type of calcium mucin and has been extensively studied, has the following characteristics: 533 amino acid residues at the N-terminal extracellular domain; 24 amino acid residues composed of highly hydrophobic molecules in the transmembrane domain; and 150 amino acid residues at the C-terminal intracellular domain (1). E-cadherin is vital for maintaining cell morphology (2), cell polarity and the connections between cells (3). Furthermore, E-cadherin is an important signal transduction molecule that participates in the transfer of information between cells (3).

Snail is a zinc-finger transcription factor. The structure of Snail consists of a variable N-terminal domain and 4–6 zinc fingers with a highly conserved C-terminal region (4). The mechanism of action for Snail involves the interaction of one of its zinc-finger areas and the E-cadherin promoter region, E-box sequence (CAGGTG), on the main chain (5). Thus, Snail becomes a transcription inhibiting protein and serves as a repressor of E-cadherin (5).

Previous studies have demonstrated that the mesenchymal-epithelial transition in tumor infiltration during migration is closely associated with the development of epithelia-mesenchymal transition (EMT), and carcinoma cells already exist in EMT (6,7). Vincent-Salomon and Thiery (8) observed that breast cancer occurs as a result of infiltration, in which the EMT and migration play important roles. Similar observations have been made in colon (9) and liver cancers (10). The indispensable transformation of epithelial cells into mesenchymal cells results in their acquisition of interstitial cell characteristics. Hence, the cells become invasive. It has been confirmed that, in tumorigenesis, EMT affects numerous transduction factors, including Snail, which represses E-cadherin expression (11); this is considered to be a key link in tumorigenesis. Snail expression is observed in tumors showing features of EMT which causes the downregulation of the E-cadherin.

Snail expression and E-cadherin repression in clear cell renal cell carcinoma (CCRCC) are rarely studied. The present study used the immunohistochemical streptavidin-peroxidase (SP) method for the combined detection of Snail and E-cadherin in CCRCC, in the normal tissues adjacent to the tumor and in normal tissues. The expression characteristics in the CCRCC were analyzed using clinical and pathological factors. Correlation between these factors would increase our understanding of the CCRCC infiltration and migration mechanism and may provide a new theoretical basis for future targeted therapy for the prevention and treatment of CCRCC infiltration and migration.

## Materials and methods

### General data

Patients aged 27–79 years (mean age: 58.41 years) were recruited from the Fuzhou General Hospital of Nanjing Military Command of Chinese PLA (Fuzhou, China) from October 2010 to June 2011. Patients were classified according to the American Joint Committee on Cancer (AJCC; 2002) TNM staging system, and the conditions were staged and graded using the Fuhrman grading system: G1, G2, G3, G4, representing high differentiation, differentiation, low differentiation, undifferentiated, respectively.. Tissue samples from 69 patients with CCRCC who had undergone radical nephrectomy were obtained preoperatively prior to the administration of any related cancer treatment. Postoperative diagnosis was performed following the pathological examination of CCRCC. Subsequently, 58 samples from normal tissues adjacent to the tumor and 10 samples from normal renal tissue were obtained and compared. This study was conducted in accordance with the Declaration of Helsinki and with approval from the Ethics Committee of Fuzhou General Hospital of Nanjing Military Command of Chinese PLA. Written informed consent was obtained from all participants.

### Immunohistochemical study

All specimens (4-μm-thick sections) were fixed with 10% formalin and embedded in paraffin, followed by preparation of 4 μm sections. 0.01 mol/l citrate buffer was added for antigen repair. The sections were examined using the SP immunohistochemical staining method. The procedure with 3,3′-diaminobenzidine staining, hematoxylin counterstaining and transparent sealing was performed according to the manufacturer’s instructions. Rabbit anti-human E-cadherin polyclonal antibody (1:100 dilution) and Rabbit anti-human Snail antibody (1:150 dilution) were provided by Beijing Biosynthesis Biotechnology Co., Ltd. (Beijing, China). Immunohistochemical SP kit was provided by Zymed Laboratories Inc. (San Francisco, CA, USA). Phosphate-buffered saline instead of primary antibody was used as negative control.

### Judgment standard

Experimental specimens were randomly observed under a high-power field microscope (magnification, ×400), and the proportion of positive cells (positivity rate) was calculated based on the positive expression (demonstrated by yellow staining) of E-cadherin and Snail in the CCRCC. The samples were determined to be positive or negative according to positive cell number: positive cell number ≥25%, positive; no positive staining or positive cell number <25%, negative (12).

### Statistical analysis

SPSS 17.0 statistical software (SPSS, Inc., Chicago, IL, USA) was used to perform the statistical analysis. The rates and relationships between two groups of variables were compared and evaluated using the χ^2^-test and Spearman rank correlation analysis, respectively. P<0.05 was considered to indicate a statistically significant result.

## Results

### Comparison of E-cadherin and Snail expression

**Figure 1:**
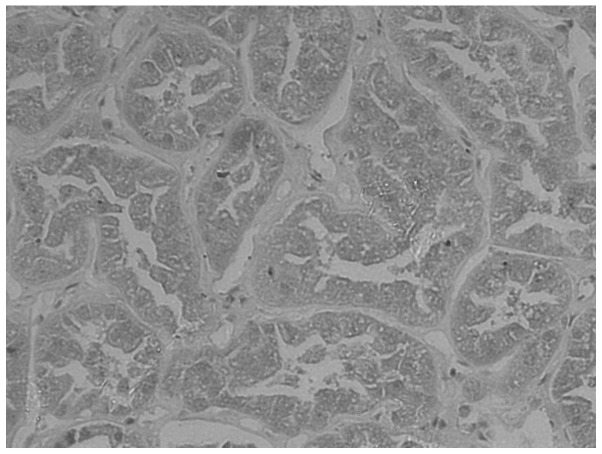
Positive expression of E-cadherin in normal renal tissues (magnification, ×400).

**Figure 2:**
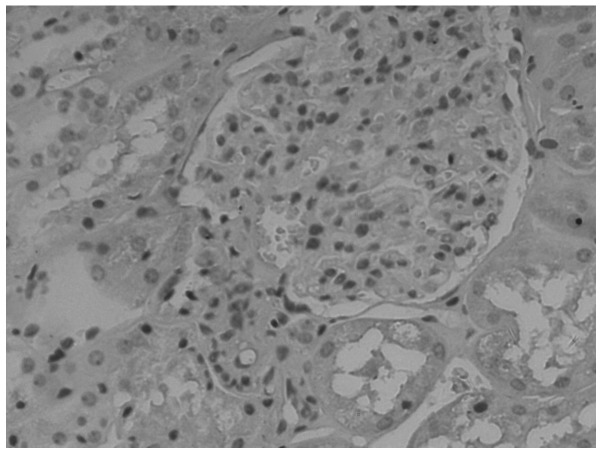
Positive expression of E-cadherin in para-cancerous mucosa (magnification, ×400).

**Figure 3:**
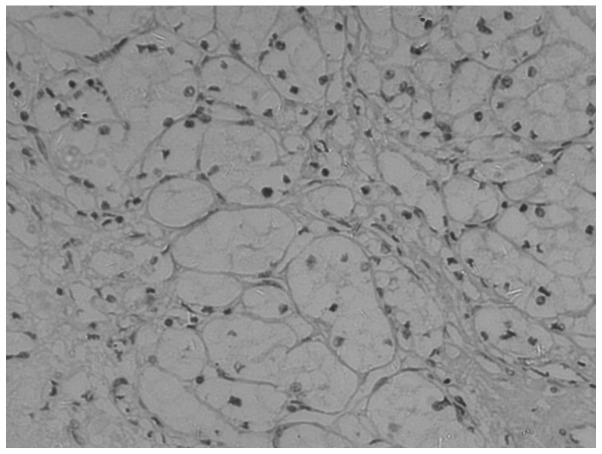
Negative expression of E-cadherin in clear cell renal cell carcinoma (magnification, ×400).

**Figure 4:**
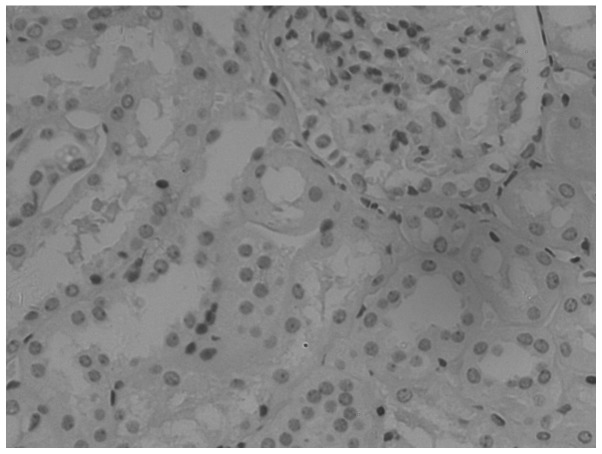
Negative expression of Snail in normal renal tissues (magnification, ×400).

**Figure 5:**
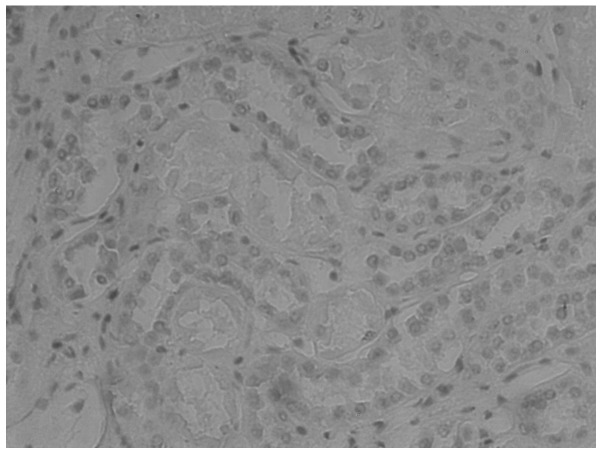
Weak positive expression of Snail in para-cancerous mucosa (magnification, ×400).

**Figure 6:**
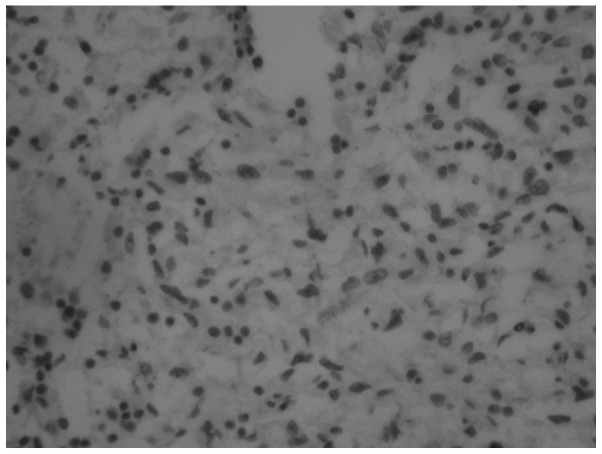
Positive expression of Snail in clear cell renal cell carcinoma (magnification, ×400).

E-cadherin expression was observed as yellow particles in the cytoplasm or membrane of normal renal cells. A low level of E-cadherin expression was detected in CCRCC. The para-cancerous and normal tissue exhibits a higher expression rate, which is located mainly in the cytoplasm and/or membranes (Figs. 1–3). The positivity rates in the tumor, normal tissues adjacent to the tumor and normal tissues were 31.88, 91.38, and 100.00%, respectively, with a statistically significant difference (P<0.001). Snail is highly expressed in CCRCC (Figs. 4–6), with positivity rates of 82.61, 43.10 and 10.00% in the tumor, normal tissues adjacent to the tumor and normal tissues, respectively. The differences in the expression rates were statistically significant (P<0.001; Table I).

### E-cadherin and Snail expression at various clinical stages

In CCRCC, the expression of E-cadherin and Snail was correlated with clinical stage and histological grade, in addition to distant and lymph node metastases (contingency coefficient r-values are shown in Table II; P<0.05). The age and gender of the patients demonstrated no significant correlation (contingency coefficient r-values are listed in Table II; P>0.05).

### Correlation of E-cadherin and Snail expression in CCRCC

Spearman rank correlation analysis revealed that the positive expression of E-cadherin is repressed as the positive expression of Snail increases; the expression of E-cadherin and Snail in the CCRCC is negatively correlated (r=0.342, P=0.004; Table III).

## Discussion

Numerous studies have shown that EMT plays an important role in the development of malignant epithelial tumors (6). A previous study has shown that during tumorigenesis and migration in the early stage of cancer, EMT results in the formation of cancer cells with specific connective tissue damage and diffused tumor cell invasion into the surrounding tissue and blood vessels (13). The EMT is also involved in embryonic neural embryo and gastrula formation and is one of the most important cell mechanisms during organ development. A number of studies have demonstrated that EMT also plays an important role in tumor development in the bladder (14), stomach (15), and colon (9), in addition to the development of malignant epithelial tumors. The mechanisms of EMT are as follows: i) Cell polarity is lost such that the expression of molecules related to cell adhesion is repressed, resulting in the destruction of the connection between specific cells. ii) The cell morphology is altered such that similar interstitial cell characteristics are obtained, accompanied by a series of changes to the molecular composition, making the cell vulnerable to strong attacks (7).

In the current study, we used a high-power microscope in order to view the cancer cells and observed that the arrangement of the cancer cells was looser compared with that of the normal renal tissue cells such that the connections between the cancer cells were not closed; the normal, organized kidney structure was lost. These observations supported the possible presence of a mechanism that leads to the formation of tumor cell connections between transformations, resulting in easy diffusion and migration. This mechanism is likely to be the EMT. Neve *et al*(16) and Charafe-Jauffret et al (17) applied the Affymetrix gene chip and proteomic principles to analyze a variety of breast cancer cell lines and observed high expression of interstitial molecular markers in the highly aggressive cell line, contrary to the low-invasive and well-differentiated cells in which the interstitial molecular markers have low expression levels or may be missing. EMT may close the connection between cells during tumor development.

Snail is a type of zinc-finger transcription factor, and its key targets in the occurrence of EMT have been identified. Snail acts through its zinc-finger area and the E-cadherin promoter E-box region (sequence CAGGTG), on the main chain, thereby repressing E-cadherin expression. The downregulation of E-cadherin directly results in alterations to the cell morphology, loss of polarity, structural instability of the organisation and the destruction of the connections between epithelial cells, resulting in the tumor cells breaking away from the original site of tumor invasion and metastasis (4). Blanco *et al*(18) found that Snail expression reduced or completely inhibited E-cadherin expression in breast ductal carcinoma and various other tumors. E-cadherin, widely distributed in epithelial cells, is stably expressed in normal epithelial cells, but its expression is repressed or completely inhibited in gastric, liver and prostate cancers in addition to various other epithelial malignant tumors (19). This is consistent with the findings in the present study in which E-cadherin expression in CCRCC was observed to be downregulated. Furthermore, we studied Snail expression in CCRCC, para-cancerous and normal tissues. A high level of Snail expression was observed in the cancerous tissue and in the majority of the histological grades, all cases of lymph node metastasis and stages.

The detection of Snail and E-cadherin expression in normal tissue, normal tissues adjacent to tumor tissue and in CCRCC using the immunohistochemical method, in addition to the internal connection of these two factors, is rarely reported. The results from the present study demonstrate a high level of Snail expression in CCRCC with a positive expression rate of 82.61% compared with those in the normal tissues near the tumor and normal tissues. The tumor stage, classification, infiltration and metastasis are correlated with the expression of Snail. Hence, Snail, to a certain extent, may reflect the ability of the tumor cells to invade. We also observed that the positive expression of Snail in CCRCC significantly repressed E-cadherin expression (P<0.05). In CCRCC, the acceleration of tumor invasion and metastasis by Snail may be achieved through the inhibition of E-cadherin expression.

In conclusion, this study evaluated the EMT and the correlation between Snail and E-cadherin protein expression in CCRCC using an immunohistochemical method. The presence of Snail and E-cadherin expression in CCRCC correlates with the CCRCC staging and grading as well as with the lymph node and distant metastasis. Therefore, the joint detection of Snail and E-cadherin expression in CCRCC may be used as a biological marker to monitor CCRCC infiltration and migration. The effect of EMT may be the inhibition of tumor infiltration or metastasis. However, the role of transformation in tumor infiltration requires further study as the linking mechanism remains unclear. Further studies are required to confirm the results of this study for the comprehensive treatment of kidney tumors and provide a theoretical basis for tumor pathogenesis in the kidney.

## Figures and Tables

**Table I:** E-cadherin and Snail expression in various tissues.

		E-cadherin		Snail	
			
Tissue type	n	+	Rate (%)	+	Rate (%)
Carcinoma	69	22	31.88	57	82.61
Tumor-adjacent	58	53	91.38	25	43.10
Normal	10	10	100.00	1	10.00

+, Positive; CCRCC, clear cell renal cell carcinoma.

**Table II:** E-cadherin and Snail CCRCC in different stages and classifications and the relationship between clinical parameters

		E-cadherin					Snail				
			
Clinical parameters	n	+	Rate (%)	χ^2^	P-value	r	+	Rate (%)	χ^2^	P-value	r
Classification				7.921	0.048	0.321			13.020	0.005	0.398
G1	11	7	63.63				5	45.45			
G2	33	10	30.30				29	87.88			
G3	20	5	25.00				18	90.00			
G4	5	0	0.00				5	100.00			
TNM				8.810	0.032	0.336			9.217	0.027	0.343
T1	36	17	47.22				25	69.44			
T2	20	4	20.00				19	95.00			
T3	10	1	10.00				10	100.00			
T4	3	0	0.00				3	100.00			
Age (years)				0.051	0.821	0.027			0.608	0.435	0.093
<58	39	12	30.77				31	79.49			
≥58	30	10	33.33				26	86.67			
Gender				0.725	0.394	0.102			0.039	0.843	0.024
Male	42	15	35.71				35	83.33			
Female	27	7	25.92				22	81.48			
Lymphaden				7.773	0.005	0.318			5.127	0.024	0.263
(−)	51	21	41.18				39	76.47			
(+)	18	1	5.56				18	100.00			
Metastases				8.972	0.003	0.328			4.035	0.045	0.235
M0	54	22	40.74				42	77.78			
M1	15	0	0.00				15	100.00			

+, positive; CCRCC, clear cell renal cell carcinoma.

**Table III:** Relationship between E-cadherin and Snail in CCRCC.

	E-cadherin expression		
		
Snail expression	Positive	Negative	Total
Positive	14	43	57
Negative	8	4	12

r=−0.342, P=0.004. CCRCC, clear cell renal cell carcinoma.
